# Opportunistic salpingectomy for ovarian cancer prevention Number 12 – 2026

**DOI:** 10.61622/rbgo/2026FPS12

**Published:** 2026-08-13

**Authors:** Agnaldo Lopes da Silva, Eduardo Batista Cândido, Jesus Paula Carvalho, Sophie Françoise Mauricette Derchain, Ricardo dos Reis

**Affiliations:** 1 Department of Gynecology and Obstetrics Faculty of Medicine Universidade Federal de Minas Gerais Belo Horizonte MG Brazil Department of Gynecology and Obstetrics, Faculty of Medicine, Universidade Federal de Minas Gerais, Belo Horizonte, MG, Brazil.; 2 Department of Gynecology and Obstetrics Faculty of Medicine Universidade Federal de Minas Gerais Belo Horizonte MG Brazil Department of Gynecology and Obstetrics, Faculty of Medicine, Universidade Federal de Minas Gerais, Belo Horizonte, MG, Brazil.; 3 Department of Obstetrics and Gynecology Faculty of Medicine Universidade de São Paulo São Paulo SP Brazil Department of Obstetrics and Gynecology, Faculty of Medicine, Universidade de São Paulo, São Paulo, SP, Brazil.; 4 Department of Obstetrics and Gynecology School of Medical Sciences Universidade Estadual de Campinas Campinas SP Brazil Department of Obstetrics and Gynecology, School of Medical Sciences, Universidade Estadual de Campinas, Campinas, SP, Brazil.; 5 Department of Gynecologic Oncology Hospital de Câncer de Barretos Barretos SP Brazil Chair, Department of Gynecologic Oncology, Hospital de Amor (Hospital de Câncer de Barretos), Barretos, SP, Brazil.

## Abstract

•Opportunistic salpingectomy (OS) is a biologically sound strategy for ovarian cancer prevention, as most high-grade serous carcinomas (HGSC) originate in the fimbrial epithelium of the fallopian tube.

•Epidemiological data and meta-analyses consistently show a substantial risk reduction (approximately 40–80%) in tubo-ovarian carcinoma following salpingectomy.

•The procedure is safe when performed during hysterectomy or benign gynecologic surgery, adding minimal operative time (10–16 minutes) without increasing perioperative complications.

•Evidence shows that it is reliable to perform OS instead of tubal ligation or fimbrectomy for permanent contraception.

•Current evidence confirms that OS does not impair short-term ovarian reserve or accelerate the onset of menopause.

•Implementation requires structured counseling on the irreversible nature of the procedure and shared decision-making.

## Recommendations

- Hysterectomy: OS is recommended during hysterectomy with ovarian preservation for benign indications in women who have completed childbearing.
- Permanent Contraception: OS should be offered as the preferred alternative to tubal ligation for women seeking permanent contraception during elective surgery.
- Other Benign Surgeries: in menopaused women and/or those who do not wish to preserve fertility. OS should be considered during surgeries for fibroids, endometriosis, or pelvic organ prolapse when adnexal access is adequate.
- Age Criteria: Patient age alone should not exclude a candidate; however, premenopausal women require detailed counseling on irreversibility.
- Contraindications: OS should not be performed in emergency settings.

## Background

Ovarian cancer remains the most lethal gynecologic malignancy worldwide, largely due to the absence of effective screening strategies and the frequent diagnosis at advanced stages. High-grade serous carcinoma (HGSC) accounts for many ovarian cancer-related deaths and is typically diagnosed at an advanced stage, contributing disproportionately to disease mortality.^(1)^

Historically, epithelial ovarian cancer was believed to originate from the ovarian surface epithelium. This concept guided decades of ovarian-focused screening strategies based on imaging and serum biomarkers, which consistently failed to demonstrate a reduction in ovarian cancer mortality.^(1,2)^

Over the past two decades, accumulating molecular, pathological, and epidemiological evidence has fundamentally revised this paradigm. Multiple studies have demonstrated that most HGSCs arise from the distal fallopian tube, particularly the fimbrial epithelium. Precursor lesions such as serous tubal intraepithelial carcinoma (STIC) and p53 signatures exhibit molecular profiles identical to those of invasive HGSC, supporting a tubal origin with subsequent spread to the ovary and peritoneal cavity.^(2,3)^

This shift in understanding has major implications for ovarian cancer prevention. By identifying the fallopian tube as the earliest site of malignant transformation, it provides a biologically coherent explanation for the failure of ovarian-focused screening and underscores the need for preventive strategies that intervene before invasive disease develops.^(1-3)^

This Position Statement aims to synthesize current evidence and support the safe, evidence-based adoption of OS in Brazilian clinical practice.

### What is the biological rationale for opportunistic salpingectomy in ovarian cancer prevention?

The biological rationale for OS is grounded in the recognition that the fallopian tube represents the primary site of origin for most HGSCs. Removal of the fallopian tubes during gynecologic surgery for benign indications, in addition to preventing endometrioid and clear cell carcinoma originating from the endometrium, directly targets the tissue at risk, interrupting the carcinogenic process at its earliest identifiable stage.^(1-4)^

Unlike screening strategies, which aim to detect established disease, OS represents a primary prevention strategy, eliminating the anatomic substrate from which most tubo-ovarian carcinomas arise. STIC lesions may remain clinically silent for prolonged periods, creating a window of opportunity for preventive surgical intervention.^(2,3)^

This biological plausibility is strongly supported by epidemiological data. Large population-based cohort studies and systematic reviews consistently demonstrate a substantial reduction in the incidence of tubo-ovarian carcinoma among women who undergo salpingectomy, particularly bilateral salpingectomy, compared with those who do not.^(4-6)^

Importantly, this reduction appears most pronounced for HGSC, aligning closely with current models of tubal carcinogenesis.

From a clinical and surgical perspective, OS is feasible, safe, and scalable. When performed during hysterectomy and in women who do not wish to preserve their fertility others gynecologic surgery for benign indications, it adds minimal operative time and does not significantly increase perioperative morbidity or short-term impairment of ovarian function.^(4,6)^

Crucially, it allows risk reduction without the endocrine and long-term health consequences associated with bilateral oophorectomy.

Taken together, the convergence of modern tumor biology, robust epidemiological evidence, and surgical feasibility provides a strong scientific foundation for OS as an effective and pragmatic ovarian cancer prevention strategy in women undergoing pelvic surgery who have completed childbearing.^(1,4-7)^

### Does opportunistic salpingectomy reduce the risk of tubo-ovarian carcinoma in the general population?

Large national cohort studies provide robust support for this association: a Swedish population-based study reported a 65% reduction (HR 0.35; 95% CI, 0.17–0.73),^(6)^ while a Danish registry analysis showed a 42% reduction (OR 0.58; 95% CI, 0.36–0.95.^(8)^

Systematic reviews and meta-analyses further reinforce these observations. A comprehensive systematic review published in 2023 reported that bilateral salpingectomy was associated with a substantial reduction in tubo-ovarian carcinoma risk, with estimates ranging from approximately 42% to 80% depending on study design and population.^(4)^

These findings are consistent across different health-care systems and surgical settings, supporting the external validity of the observed risk reduction.

Based on a structured review of the available evidence and a formal consensus process, the European Society of Gynaecological Oncology concluded that OS is significantly associated with a lower incidence of tubo-ovarian carcinoma and should be considered during gynecologic surgery for benign indications and selected nongynecologic pelvic procedures.^(1)^

Tubal ligation has a protective effect specifically against endometrioid and clear cell carcinomas of the ovary, which support the theory that these tumors may be related to retrograde menses of endometrial cells.^(9)^

Importantly, current evidence suggests that this protective effect is most pronounced for high-grade serous carcinoma and related tubo-ovarian malignancies and does not uniformly extend to all ovarian cancer histologic subtypes.^(10)^

Taken together, these data provide consistent and biologically plausible evidence that OS reduces the risk of tubo-ovarian carcinoma in the general population, supporting its adoption as a preventive strategy in appropriate clinical contexts.

### What is the magnitude and consistency of cancer risk reduction associated with opportunistic salpingectomy?

Importantly, the direction and magnitude of risk reduction are remarkably consistent, despite heterogeneity in study methodology, follow-up duration, and comparator groups. The protective effect appears most pronounced for high-grade serous carcinoma, aligning with the fallopian tube as the principal site of origin of this subtype.^(1)^

However, like tubal ligation, it also reduces clear cell carcinoma and endometriosis.^(9)^

Although the absolute reduction in cancer incidence is modest due to the rarity of the disease, the consistency and magnitude of relative risk reduction support a meaningful population-level preventive impact.

### Is opportunistic salpingectomy a safe procedure when added to benign gynecologic surgery?

Available evidence from randomized controlled trials, large cohort studies, and systematic reviews demonstrates that OS performed during benign gynecologic surgery is not associated with an increased risk of intraoperative or short-term postoperative complications when compared with hysterectomy or sterilization alone.^(1,5,11,12)^

Across studies, the addition of salpingectomy is consistently associated with a small increase in operative time, generally ranging from 10 to 16 minutes, without clinically meaningful differences in estimated blood loss, need for blood transfusion, length of hospital stay, or rates of infection, readmission, or reoperation.^(1,11,12)^

These findings are reproducible across different surgical approaches and health-care settings.

Large population-based cohort studies and meta-analyses further confirm the absence of increased rates of major or minor surgical complications associated with OS.^(5,11,12)^

Although some observational data suggest a slight increase in short-term analgesic use or self-reported menopausal symptoms, these findings are inconsistent and not accompanied by higher rates of objective postoperative morbidity.^(5,13)^

Based on a comprehensive review of the available evidence and expert consensus, international societies conclude that OS is a safe adjunct to benign gynecologic surgery and that its performance should not require modification of the planned surgical route. Patients should be counseled regarding the modest increase in operative time and the current evidence supporting surgical safety.^(1,12)^

### Does the surgical route influence the feasibility or safety of opportunistic salpingectomy?

The feasibility of OS varies according to the surgical route, while overall safety remains preserved when the procedure is performed in appropriate clinical settings and by experienced surgeons.

Laparoscopic and robotic approaches provide optimal visualization of the adnexa and are associated with high feasibility rates for OS. These minimally invasive routes allow reliable identification and removal of the fallopian tubes across a wide range of pelvic anatomies and are considered the preferred approaches when available.^(1,11)^

Open abdominal surgery (laparotomy) similarly allows safe access to the fallopian tubes and does not represent a technical limitation for OS when performed as part of planned pelvic procedures.^(1)^

In contrast, vaginal hysterectomy presents greater technical constraints, primarily due to limited visualization and access to the adnexa. Reported feasibility rates range from approximately 75% to 88% and are influenced by uterine mobility, pelvic anatomy, and the presence of adhesions. In such cases, OS may not be feasible without increasing surgical complexity, and intraoperative judgment is required.^(12,14)^

Opportunistic salpingectomy may also be considered during selected nongynecologic abdominal procedures, provided adequate adnexal exposure is achieved. However, extensive adhesions, distorted pelvic anatomy, or limited access to the fallopian tubes represent the main factors limiting feasibility in these settings.^(1)^

International expert consensus emphasizes that the decision to perform OS should not modify the initially planned surgical route. When technical limitations or altered pelvic anatomy increase operative risk, the procedure should be safely omitted without compromising the primary surgical objective.^(1)^

### Does opportunistic salpingectomy impair short-term ovarian function?

Current evidence demonstrates that OS does not impair short-term ovarian function. Randomized controlled trials, systematic reviews, and meta-analyses consistently show no significant differences in ovarian reserve markers, including anti-Müllerian hormone (AMH), follicle-stimulating hormone (FSH), antral follicle count (AFC), and estradiol levels, when comparing women undergoing hysterectomy or sterilization with or without OS.^(1,15-17)^

These findings are consistent across different surgical indications and approaches.

Recent systematic reviews and meta-analyses confirm that short-term postoperative changes in ovarian reserve markers after OS are comparable to those observed after hysterectomy alone, supporting the absence of a clinically meaningful impact on ovarian function in the immediate and short-term postoperative period.^(1,16,17)^

Although a theoretical concern exists regarding potential disruption of ovarian blood supply, available clinical data does not demonstrate functional impairment of the ovary in the short term. On this basis, international expert consensus considers OS to be ovarian-function–sparing in the short term when appropriately performed.

### Is there evidence that opportunistic salpingectomy affects age at menopause or long-term ovarian function?

There is currently no evidence that OS affects age at menopause or leads to clinically relevant long-term impairment of ovarian function. Available data from population-based cohort studies, systematic reviews, and meta-analyses do not demonstrate an increased risk of earlier menopause, premature ovarian insufficiency, or higher use of menopausal hormone therapy among women who underwent OS compared with appropriate control groups.^(1,11,16,17)^

Registry-based cohort study with follow-up extending beyond five years show no significant differences in time to menopause, menopause-related physician visits, or initiation of hormone therapy after OS performed during hysterectomy or as a method of sterilization.^(5)^

These findings provide indirect but reassuring evidence regarding long-term ovarian endocrine function.

Systematic reviews and meta-analyses assessing ovarian reserve markers also support the absence of long-term harm, although most studies include follow-up periods limited to the short or intermediate term.^(11,16,17)^

While these markers are not direct surrogates for age at menopause, their stability over time is consistent with preserved ovarian function.

Nevertheless, long-term evidence remains limited, particularly regarding outcomes beyond 10 years and in younger premenopausal women. As a result, a small effect on the timing of menopause or subtle long-term ovarian changes cannot be completely excluded at present. Ongoing prospective studies, including the STOPOVCA trial, have been specifically designed to address this knowledge gap, but definitive results are not yet available.^(18)^

### In which clinical scenarios should opportunistic salpingectomy be routinely recommended?

Opportunistic salpingectomy should be routinely recommended during hysterectomy with ovarian preservation performed for benign indications in women who have completed childbearing, provided there are no technical contraindications. This clinical scenario represents the most consistent and evidence-supported indication, combining high feasibility, established surgical safety, and a meaningful potential reduction in the risk of tubo-ovarian carcinoma.^(1,7,19)^

International and national expert consensus statements recommend that all eligible women undergoing pelvic surgery be informed about the option of OS, including during selected nongynecologic abdominal procedures when access to the fallopian tubes is adequate. However, the procedure should not be performed if extensive adhesions, distorted pelvic anatomy, or other technical factors substantially increase surgical risk.^(1,17,19)^

In all clinical scenarios, counseling must include discussion of permanent loss of tubal fertility, expected cancer risk reduction, and remaining long-term uncertainties, without altering the originally planned surgical route.^(7)^

Clinical decision-making for OS is summarized in Figure 1.

**Figure 1:**
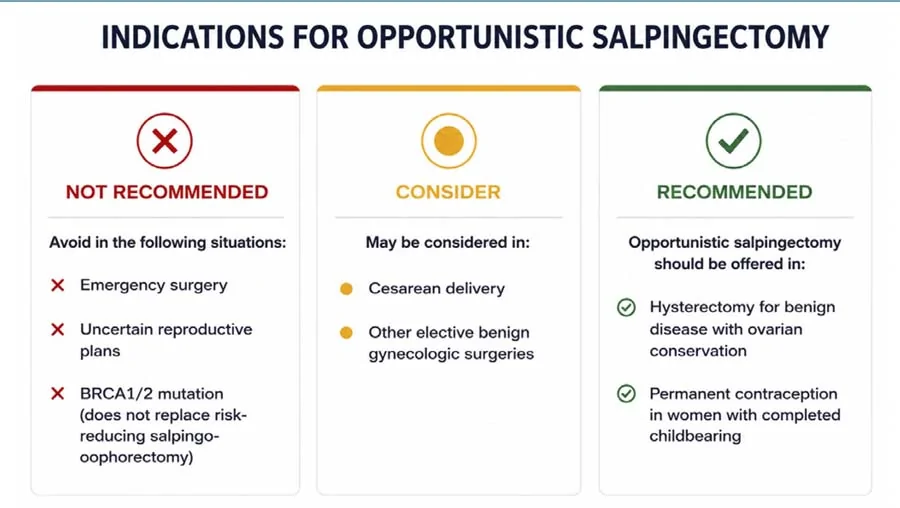
Clinical decision framework for opportunistic salpingectomy

### Should opportunistic salpingectomy replace tubal ligation as the preferred method of permanent contraception?

Opportunistic salpingectomy may replace tubal ligation as the preferred method of permanent contraception in women who have completed childbearing, provided that individualized counseling and shared decision-making are ensured. This recommendation is grounded in the current understanding of tubo-ovarian carcinogenesis, in which the fallopian tube, particularly the fimbrial end, is recognized as the predominant site of origin of HGSC.^(1,7,11)^

International societies and expert groups increasingly support OS as an alternative to tubal ligation, based on its comparable contraceptive efficacy and the additional benefit of reducing the lifetime risk of tubo-ovarian carcinoma.^(1,11,20)^

In a national Delphi study among Brazilian experts, there was strong consensus that OS should be discussed and preferentially considered over tubal ligation for definitive contraception in women with completed family planning, particularly when performed during cesarean delivery or elective gynecologic surgery.^(7)^

Current scientific literature suggests that salpingectomy offers contraceptive effectiveness equivalent to tubal ligation, with similar rates of perioperative complications and no clinically relevant short-term impact on ovarian function.^(5-8,21,22)^

Observational studies and randomized data indicate that the procedure may be associated with a modest increase in operative time but does not significantly increase blood loss, hospital stay, infection, or major surgical morbidity when compared with tubal occlusion.^(21,22)^

Health economic evaluations further suggest that OS is cost-effective compared with tubal ligation in high-volume clinical scenarios such as cesarean delivery and postpartum sterilization, owing to its potential to prevent future ovarian cancer cases and related mortality.^(23)^

However, salpingectomy is not more cost-effective than vasectomy when male sterilization is an acceptable option, and this alternative should be included in comprehensive contraceptive counseling.^(24)^

Despite these advantages, there is no absolute consensus that OS should universally replace tubal ligation in all settings. Barriers to widespread adoption include increased technical complexity in some clinical scenarios, variability in surgical expertise, logistical constraints, and the absence of definitive long-term data on ovarian function and age at menopause.^(7,11,25)^

In addition, concerns regarding potential regret, particularly among younger women, reinforce the need for careful patient selection and counseling.^(7,25)^

Accordingly, shared decision-making is essential. Counseling should explicitly address the permanent nature of the procedure, the expected reduction in ovarian cancer risk, the equivalence in contraceptive efficacy compared with tubal ligation, and the remaining uncertainties regarding long-term ovarian outcomes. Opportunistic salpingectomy should not be performed in emergency situations or when future reproductive intentions are unclear.^(7,11)^

### Can opportunistic salpingectomy replace risk-reducing salpingo-oophorectomy for ovarian cancer prevention?

Risk-reducing salpingo-oophorectomy (RRSO) remains the standard of care for women at increased hereditary risk, particularly carriers of pathogenic variants in *BRCA1* or *BRCA2*. By removing both the fallopian tubes and ovaries, RRSO provides the most effective reduction in the risk of ovarian, fallopian tube, and primary peritoneal cancer, with estimates of approximately 70–85% risk reduction, and is also associated with a reduction in overall mortality and breast cancer risk in this population.^(19,26,27)^

Although contemporary models of tubo-ovarian carcinogenesis support the fallopian tube as the predominant site of origin of HGSC, salpingectomy alone leaves the ovaries intact. Consequently, a residual risk of ovarian or peritoneal carcinogenesis persists, and OS does not address hormone-driven breast cancer risk in genetically predisposed women.^(19,26,27)^

For these reasons, international expert consensus consistently emphasizes that OS should not be considered an equivalent substitute for RRSO in high-risk women.^(1,19,27)^

The strategy of salpingectomy with delayed oophorectomy has been proposed as a potential alternative for selected high-risk women who decline immediate oophorectomy, primarily to mitigate the adverse consequences of premature surgical menopause. While pilot studies and modeling analyses suggest potential quality-of-life benefits and theoretical preservation of cancer risk reduction if oophorectomy is performed within a defined time window, there is currently no direct clinical evidence demonstrating oncologic equivalence to immediate RRSO.^(19,27,28)^

As such, this approach remains investigational and should be limited to research settings or highly selected cases with careful counseling.

### Should patient age influence the recommendation for opportunistic salpingectomy?

Patient age alone should not be used as an exclusion criterion for recommending OS. Current evidence and expert consensus indicate that the procedure may be considered across a wide age range in women undergoing gynecologic or selected abdominal surgery for benign indications, provided that childbearing is complete and ovarian preservation is intended.^(1,7,11)^

Available data demonstrate that OS is safe in premenopausal women, with no clinically relevant impact on short-term ovarian function and no robust evidence of earlier menopause or long-term ovarian impairment to date.^(1,11,16)^

Therefore, chronological age, should not, by itself, represent a barrier to offering OS when technical conditions are favorable and surgical risk is acceptable.

However, women of reproductive age and premenopausal women require individualized counseling, given the irreversible nature of salpingectomy and the remaining uncertainties regarding very long-term ovarian endocrine outcomes. A national Delphi study among Brazilian experts reached strong consensus that age should not be an absolute limiting factor but emphasized that younger women must receive detailed counseling addressing fertility irreversibility, expected oncologic benefit, and the current limits of long-term evidence.^(7)^

For younger premenopausal women, counseling should explicitly include discussion of:

- permanent loss of tubal fertility.
- absence of demonstrated short- and mid-term harm to ovarian reserve.
- lack of definitive data on ovarian function and age at menopause beyond long-term follow-up; and alternative options for permanent contraception, including male sterilization when appropriate.^(1,7,11)^

In postmenopausal women undergoing pelvic surgery with ovarian preservation, OS may be offered without age-related restrictions, as concerns regarding fertility and long-term endocrine effects are no longer applicable, and the potential benefit in cancer risk reduction remains biologically plausible.^(1)^

### What are the key elements of preoperative counseling and informed consent for opportunistic salpingectomy?

Preoperative counseling and explicit informed consent are essential prerequisites for OS. Counseling must ensure that the woman clearly understands the rationale, benefits, limitations, and irreversible nature of the procedure before a shared decision is made.^(1,7,19)^

The consent process should include a clear explanation of the expected benefits, particularly the reduction in the lifetime risk of tubo-ovarian carcinoma, while emphasizing that risk is reduced but not eliminated.^(1,19)^

Women should also be informed that current evidence demonstrates no relevant increase in perioperative complications and no impairment of short-term ovarian function, but that the procedure is performed for cancer prevention rather than for immediate clinical benefit.^(1,11,19)^

Irreversibility is a mandatory component of counseling. Opportunistic salpingectomy results in permanent loss of tubal fertility and should only be offered to women with completed childbearing or a definitive desire for permanent contraception. A national Delphi study among Brazilian experts strongly emphasized that explicit discussion of irreversibility and documentation of patient understanding are essential to ethical implementation.^(7)^

Counseling must also address the existing uncertainties regarding long-term outcomes, particularly the limited availability of data on ovarian endocrine function and age at menopause beyond long-term follow-up. Although current evidence is reassuring, women, especially those who are younger and premenopausal, should be informed that very long-term effects cannot yet be completely excluded.^(1,7,19)^

Discussion of alternatives is another key element of informed consent. These include tubal ligation, male sterilization, or no surgical contraception, depending on the clinical context and patient preferences. Importantly, the decision to perform OS should not alter the planned surgical route or increase operative risk.^(1,19)^

Structured counseling, supported by decision aids and written educational materials, has been shown to improve patient understanding and alignment between medical recommendations and individual values. Both international consensus statements and Brazilian expert opinion recommend that consent should never be obtained under time pressure or in emergency situations (such as ruptured ovarian cysts, pelvic inflammatory disease, ectopic pregnancy, or adnexal torsion), allowing adequate time for reflection and shared decision-making.^(7,19,29)^

Informed consent must be explicit, structured, and individualized, addressing benefits, irreversibility, alternatives, and remaining long-term uncertainties.^(1,7,19)^

Key domains and essential elements for preoperative counseling and informed consent are summarized in Table 1.

**Table 1:** Key elements of counseling and informed consent for opportunistic salpingectomy

Counseling domain	Essential points to address
Cancer risk reduction (relative, not absolute)	OS is associated with a significant reduction in the risk of tubo-ovarian carcinoma, particularly high-grade serous carcinoma, but does not eliminate cancer risk. Residual risk persists due to the presence of ovaries and the possibility of peritoneal carcinogenesis.
Irreversibility of fertility loss	Salpingectomy results in permanent loss of tubal fertility and should only be offered to women with completed childbearing or a definitive decision for permanent contraception. Reversal is not possible.
Alternatives (tubal ligation, vasectomy)	Alternative options for permanent contraception should be discussed, including tubal ligation and male sterilization (vasectomy), with comparison of effectiveness, risks, and lack of ovarian cancer risk reduction associated with some alternatives.
Ovarian function and menopause	Current evidence shows no clinically relevant short-term impairment of ovarian function after OS. Available data does not demonstrate an increased risk of earlier menopause, although long-term data remain limited.
Long-term uncertainties	Patients should be informed that, while existing evidence is reassuring, very long-term effects on ovarian endocrine function and age at menopause are not fully established, particularly in younger premenopausal women.
Situations where OS should not be performed	OS should not be performed in emergency settings, in women with uncertain reproductive plans, in the absence of explicit informed consent, or when technical or clinical factors substantially increase surgical risk.

### In which situations should opportunistic salpingectomy not be performed?

Opportunistic salpingectomy should not be performed in situations where adequate informed consent cannot be obtained, when future reproductive wishes are uncertain, or when the procedure would increase surgical risk without proportional benefit. These circumstances represent relative contraindications and require prioritization of patient safety and autonomy.^(1,7,19)^

Emergency surgery constitutes a clear contraindication to OS. In urgent or emergent settings, there is insufficient time for structured counseling and shared decision-making, and consent obtained under these conditions does not meet ethical standards for an irreversible preventive procedure. Both international consensus statements and Brazilian expert opinion emphasize that OS should not be proposed or performed in emergency contexts.^(1,7)^

Uncertain or uncompleted reproductive plans are another key contraindication. Because OS results in irreversible loss of tubal fertility, it should not be performed in women who have not clearly completed childbearing or whose reproductive intentions are not firmly established. The Brazilian Delphi study highlighted strong consensus that reproductive uncertainty should preclude the procedure, even when salpingectomy could be technically feasible.^(7)^

Increased surgical risk related to anatomical or clinical factors represents an important relative contraindication. These include extensive pelvic adhesions, marked distortion of pelvic anatomy, severe endometriosis, prior multiple abdominal surgeries, or any condition in which salpingectomy would substantially increase operative complexity or the risk of intraoperative complications. In such cases, the procedure should be omitted, and the originally planned surgery should proceed without modification.^(1,19)^

Absence of explicit informed consent is an absolute contraindication. Opportunistic salpingectomy should only be performed when the patient has received structured preoperative counseling, understands the irreversible nature of the procedure, its expected benefits, limitations, and remaining long-term uncertainties, and has provided documented consent. This principle is consistently reinforced by international guidelines and national expert consensus.^(1,7,19)^

Opportunistic salpingectomy should not be performed in emergency settings, in women with uncertain reproductive wishes, in the absence of explicit informed consent, or when technical or clinical factors significantly increase surgical risk. In these situations, patient safety, reproductive autonomy, and ethical standards must take precedence over potential preventive benefit.^(1,7,19)^

### What are the main barriers to the adoption of opportunistic salpingectomy in clinical practice?

The adoption of OS in routine clinical practice remains heterogeneous and is limited by a combination of professional, educational, organizational, and cultural barriers, particularly in the Brazilian context.

Professional barriers include variability in clinician awareness, acceptance, and confidence in recommending OS. Uncertainty regarding long-term ovarian outcomes, concerns about irreversibility, and perceived technical difficulty, especially in vaginal surgery or in patients with complex pelvic anatomy, contribute to inconsistent implementation. A national Delphi study among Brazilian experts identified lack of uniform clinical practice and persistent concerns about long-term safety as key limiting factors, despite overall agreement on the potential benefits of the procedure.^(1,7)^

Educational barriers are closely related and include insufficient training on the fallopian tube as the primary site of origin of HGSC, as well as limited exposure to OS techniques during residency and continuing medical education. The absence of standardized counseling tools and decision aids further hampers consistent patient communication and shared decision-making.^(7,29,30)^

Organizational and health system barriers play a major role in limiting uptake. These include the lack of formal inclusion of OS in institutional protocols and national guidelines, limited access to minimally invasive surgical training, time constraints in high-volume surgical settings, and variability in resource availability between public and private health systems. Regional disparities within Brazil further exacerbate inequities in access to elective gynecologic surgery and preventive strategies.^(1,7,31)^

Cultural and patient-related barriers include reluctance to remove macroscopically normal organs, fear of permanent sterilization and future regret, limited awareness of ovarian cancer severity, and insufficient understanding of the preventive rationale of salpingectomy. Both patient and provider discomfort in discussing cancer prevention during surgery for benign indications has been identified as a relevant obstacle.^(7,30)^

In Brazil, these barriers are amplified by the absence of long-standing national guidance, uneven dissemination of emerging evidence, and structural limitations within the health system. Overcoming these challenges requires multilevel strategies, including professional education, incorporation OS into national and institutional guidelines, development of culturally appropriate counseling materials, and promotion of shared decision-making as standard practice.^(1,7,30)^

## Final Considerations

Opportunistic salpingectomy has consolidated its role as a safe and evidence-based strategy for the primary prevention of tubo-ovarian carcinoma, particularly HGSC, now recognized to originate predominantly in the fallopian tube. Large population-based studies, systematic reviews, and meta-analyses consistently demonstrate a meaningful reduction in ovarian cancer risk, without a relevant increase in perioperative morbidity or short-term impairment of ovarian function when the procedure is performed during benign gynecologic surgery in women who have completed childbearing.^(1,4,6)^

Current evidence does not indicate a clinically significant impact on ovarian reserve or age at menopause in the short to intermediate term.^(1,4,5)^

However, as long-term data beyond five to ten years remain limited, especially in younger premenopausal women, a small residual uncertainty persists. For this reason, OS should be offered within a framework of structured preoperative counseling and shared decision-making, clearly addressing benefits, irreversibility, and remaining knowledge gaps.^(1,4,5)^

Risk-reducing salpingo-oophorectomy continues to be the standard of care for women carrying pathogenic variants in BRCA1 or BRCA2. To date, the literature consistently underscores that opportunistic salpingectomy should not be regarded as an equivalent alternative to RRSO in women at high risk.^(1,19,27)^

The translation of this evidence into routine clinical practice requires coordinated professional guidance, standardized counseling, and ongoing education. In Brazil, FEBRASGO plays a central role in promoting the safe, ethical, and evidence-based adoption of OS by supporting professional training, fostering consensus, and encouraging alignment between international evidence and national clinical practice. Such leadership is essential to ensure equitable implementation across diverse healthcare settings and to stimulate data collection that may further refine future recommendations.^(7)^

In summary, OS represents a valuable preventive intervention with a favorable benefit–risk profile, whose incorporation into clinical practice should be guided by high-quality evidence, individualized counseling, and institutional support.
